# HaVec: An Efficient de Bruijn Graph Construction Algorithm for Genome Assembly

**DOI:** 10.1155/2017/6120980

**Published:** 2017-08-27

**Authors:** Md Mahfuzer Rahman, Ratul Sharker, Sajib Biswas, M. Sohel Rahman

**Affiliations:** Department of CSE, BUET, ECE Building West Palasi, Dhaka 1205, Bangladesh

## Abstract

**Background:**

The rapid advancement of sequencing technologies has made it possible to regularly produce millions of high-quality reads from the DNA samples in the sequencing laboratories. To this end, the *de Bruijn graph* is a popular data structure in the genome assembly literature for efficient representation and processing of data. Due to the number of nodes in a de Bruijn graph, the main barrier here is the memory and runtime. Therefore, this area has received significant attention in contemporary literature.

**Results:**

In this paper, we present an approach called HaVec that attempts to achieve a balance between the memory consumption and the running time. HaVec uses a hash table along with an auxiliary vector data structure to store the de Bruijn graph thereby improving the total memory usage and the running time. A critical and noteworthy feature of HaVec is that it exhibits no false positive error.

**Conclusions:**

In general, the graph construction procedure takes the major share of the time involved in an assembly process. HaVec can be seen as a significant advancement in this aspect. We anticipate that HaVec will be extremely useful in the de Bruijn graph-based genome assembly.

## 1. Background

The rapid advancement of the next-generation sequencing technologies has made it possible to regularly produce numerous reads from the DNA samples in the sequencing laboratories. In particular, the number of reads now is in the range of hundreds of millions. Hence, the current challenges include efficient processing of this data which may reach even a couple hundred GB. To this end, the *de Bruijn graph* is a popular data structure in the genome assembly literature for efficient representation and processing of data. In a de Bruijn graph, the nodes represent the distinct *k*-mers that occur in the reads and there exists an edge between the two nodes if there is a (*k*–1)-length overlap between the suffix and prefix of the corresponding *k*-mers, respectively. Because there could be a huge number of nodes in a de Bruijn graph, the researchers are motivated to focus on devising a compact representation of this graph. Example of such works includes but are not limited to [1–8].

The *Bloom filter* is a popular data structure that can represent a set and is capable of testing whether a given element is present or not there. And it can do this efficiently both in terms of memory and speed. The base data structure of a Bloom filter consists of an *m*-bit array, initialized to zero. It further uses *h* hash functions. To insert or test the membership of an element, a total of *h* array positions are computed using each of the *h* hash functions. To insert, all corresponding positions in the bit array are set to 1. Similarly, the membership operation returns yes if and only if all of these bit positions have 1 (i.e., are set). Note that the Bloom filters are probabilistic data structures: a negative response to a membership test for an element ensures that the element is definitely absent; however, a positive response cannot certainly indicate the presence of the element in the set. So, even if a Bloom filter membership test returns true, the element may not in fact be present in the set. Such a positive response is referred to as a “false positive.”

Designing lightweight implementations of de Bruijn graphs has been the focus of attention in recent times. For example, minimum-information de Bruijn graphs, pioneered by [3], ensure its lightweight by not recording read locations and paired-end information. A distributed de Bruijn graph is implemented by [4] which reduces the memory usage per node. On the other hand, Conway and Bromage [5] have proposed storing an implicit, immutable graph representation by applying sparse bit array structures. In these methods, portions of the de Bruijn graph are greedily extended to compute local assemblies around sequences of interest, and these methods use negligible memory. Interestingly, Ye et al. [6] proved that a graph roughly equivalent to the de Bruijn graph can be obtained by storing only one out of *g* nodes (10 ≤ *g* ≤ 25).

Pell et al. [7] have employed a Bloom filter to devise the probabilistic de Bruijn graph. Using their method, the graph encoding can be achieved with as little as 4 bits per node. However, the inherent limitation of the Bloom filter is that it can report false positive results in the introduction of false nodes and false branching in their approach. Still, it can be shown that the global structure of the graph can be approximately preserved, up to a certain false positive rate. Notably, in [7], we do not find the authors to perform the assembly directly by traversing the probabilistic graph. Instead, the graph has firstly been used to partition the set of reads into smaller sets, and subsequently a classical assembler has been used for assembly purposes.

Recently, Chikhi and Rizk [8] have again proposed a Bloom filter based on a new encoding of the de Bruijn graph. They have introduced an additional structure that is instrumental in removing critical false positives. One drawback of their approach is the use of auxiliary memory, that is, its strong dependence on the free space in the hard disk. This in fact can affect the performance of their approach severely. In particular, this is clearly evident when the number of unique *k*-mers in a file skyrockets. For example, when the number of unique *k*-mers in a file becomes 2 × 10^9^, it takes more than 10 hours to complete the critical false positive calculation. To summarize, their approach, in addition to the RAM usage, requires the total free hard disk space to be used over and over again. This in the end affects the runtime and it becomes prohibitively high. Another limitation of this approach is that it cannot handle the situation when the *k*-mers are of even length.

According to the present state of the art, memory-efficient Bloom filter representations of de Bruijn graphs have two critical issues, namely, the high running time and the task of false positive computation. On the other hand, other traditional approaches that do not have these issues need much higher memory.

In this paper, we make an effort to alleviate these problems. In particular, we present a new algorithm based on *hashing* and *auxiliary vector data structures* and call this algorithm *HaVec*. The key features of HaVec are as follows which can be seen as the main contributions in this paper:

1. HaVec introduces a novel graph construction approach that has all three desired properties: it is error free, its running time is low, and it is relatively memory efficient and hence requires sufficiently low memory.
2. It introduces the idea of using a hash table along with an auxiliary vector data structure to store the *k*-mers along with their neighbour information.
3. It constructs such a graph representation that generates no false positives. As a result, only true neighbours are found for traversing the whole graph.

We note that some preliminary results of this research work were presented at the 17th International Conference on Computer and Information Technology (ICCIT 2014) [9].

## 2. Methods

### 2.1. General Overview

Let us consider the genome assembly process when a de Bruijn graph is used. Because of the high memory requirement, traditional graph representation approaches do not scale well. This is specially true in case of large graphs having millions of nodes and edges. A Bloom filter can offer a memory-efficient alternative. In this option, edge is not stored explicitly; rather a present bit is used for every node. The procedure is well known and briefly described below for completeness. For each node in the graph, a hash value is produced, which along with the table size produces an index in the table. The most popular and easy method to produce this index is to divide the hash value by the table size to get the remainder. Now, if the node is present, the corresponding index as calculated above is set to 1. Similarly, to check the presence (absence) of a node in the graph, we do the same calculation and simply check whether the corresponding index is 1 (0). At this point, recall that a Bloom filter may produce false positives. Hence, if the corresponding index is 0, then the node is definitely absent; otherwise, the node is possibly present.

Now the question is how can we compute the edges? Again, the procedure is simple. Recall that a node corresponds to a *k*-mer. So, from a node (say *x*), all possible neighbours can be easily generated. Now we can easily check whether a generated possible neighbour (say *y*) is indeed present or not in the same way described above. And if *y* is absent in the Bloom filter, we can decide that the edge (*x*, *y*) is definitely absent in the graph; otherwise, the edge is possibly present there.

Now the problem of using the Bloom filter to represent the graph lies in the probability that more than one node may generate the same index: when divided by the table size and hash values of more than one node may produce the same remainder. So, there is a chance for a false edge to be created in the graph if a neighbour node is generated falsely; that is, if the corresponding bit is set due to a different node generating the same reminder. This is why we may have false positives when using a Bloom filter.

If the false positives are eliminated, then, the Bloom filter will undoubtedly be one of the best candidates (if not the best) to represent a de Bruijn graph. Note that an increase in the table size of a Bloom filter surely decreases the false positive rate; however, it will never become zero. In this paper, we present a crucial observation to tackle this issue: even if the same reminder is produced from more than one node following the abovementioned division operation (i.e., (hash value)/(table size)), the quotient for each division operation must be different. So, if two nodes are pointing to the same index in the hash table, by examining the respective quotient values, we can easily verify which one is falsely generated and which one is indeed the real one. This works like a fairy tale! However, there is a catch: now, for each index in the table, we have to keep track of a mapping between hash values and quotients.

Our approach is quite simple and described below. We use a total of *h* different hash functions (say *H*_*i*_, 1 ≤ *i* ≤ *h*). So for each node, this allows us to produce a total of *h* hash values. At first, we make an attempt to store the node using the index generated by *H*_1_. If that fails, that is, if some other node has already occupied it, we use *H*_2_ and so on. However, it may very well happen that all *H*_*i*_ and 1 ≤ *i* ≤ *h* fail to provide a free index. In that case, being out of options, we have to resort to our auxiliary vector data structure. We now use the index value generated by the last hash function, *H*_*h*_, to select a position in the vector data structure. Note that the same problem of multiple index values pointing to the same position can happen here as well. This is handled by maintaining a list of indices in that position. A (second level) vector structure is maintained for a particular index of that list, where all the collided nodes on that index are stored. For a detailed description please refer to Section 2.3.

### 2.2. de Bruijn Graphs, Hash Tables, and Auxiliary Vector Structures

As has been mentioned above, HaVec does not maintain an explicit graph structure; rather, it uses the *k*-mer's information to construct the de Bruijn graph. And it stores the information of the *k*-mers using the hash table and if needed using the auxiliary vector data structures. Given a *k*-mer (i.e., a node), HaVec can generate its correct neighbours simply by examining its neighbour bits. In what follows, we will describe the procedure in detail.

#### 2.2.1. Hash Table Structure

HaVec uses hashing for faster access. In the hash table, for each index, HaVec uses 40 bits, that is, 5 bytes of memory as will be evident shortly (please see also Table 1).

1. Because we are working on DNA sequences, each node (i.e., *k*-mer) cannot have more than four neighbouring *k*-mers. To compute a possible neighbour of a given *k*-mer, we just need to remove its first symbol after appending it to one of the four nucleotides. Now, there are a total of 16 possible ways one *k*-mer can have neighbours:
  - It can have no neighbours (we have only one possibility).
  - Or it can have only one neighbour (we have 4 possibilities).
  - Or it can have only 2 neighbours (we have $\left(\begin{array}{c} 4\\ 2 \end{array}\right)=6$ possibilities).
  - Or it can have 3 neighbours (we have $\left(\begin{array}{c} 4\\ 3 \end{array}\right)=4$ possibilities).
  - Or it can have all 4 neighbours (we have only one possibility).

Hence, HaVec employs 4 bits for this purpose, where a particular bit corresponds to a particular nucleotide.

(2) HaVec uses 3 bits to keep track of the hash functions thereby accommodating a maximum of 8 hash functions (in this setting).
(3) The quotient value therefore can be stored in the remaining 33 bits.

#### 2.2.2. Auxiliary Vector Data Structures

**Figure 1:**
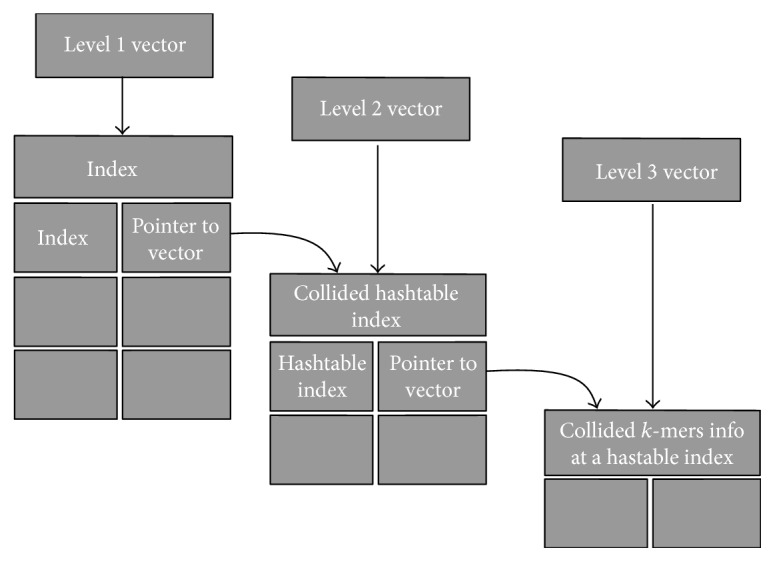
Three levels of vectors used in our approach.

HaVec employs an auxiliary vector data structure as shown in Figure 1. As can be seen, there exist three levels of indirection in the vector data structure. Each entry in the first level keeps a pointer to a list containing (one or more) hash table indices; this is the second level (level 2) vector structure. Each entry in the Level 2 vector corresponds to a particular hash table index and keeps track of (by pointing to) all the collided *k*-mer's information pertaining to that particular hash table index. Finally, the level 3 vector, which is pointed to from a second level vector entry, keeps the record of all collided *k*-mers for a particular hash table index.

#### 2.2.3. Size of the Quotient Value

In order to represent the hash value of a *k*-mer, HaVec requires 2*k* bits. Since we have 33 bits to store a quotient value, we need to ensure that the total number of hash indexes, that is, the hash table size, is at least 2^(2*k-*33)^. This is because the quotient value is computed by dividing the hash value by the table size. We illustrate this with the help of an example. Suppose that the value of *k* is 32. Then HaVec requires 2*k* bits, that is, 64 bits to represent the hash values. Clearly, the maximum possible hash value would be 2^64^–1. Now, the minimum hash table size of 2^64-33^ or 2^31^ implies that the maximum quotient value can be 2^33^–1 requiring 33 bits of storage. Clearly, the minimum hash table size is dependent on the value of *k*: for smaller *k*, it will decrease. For example, if *k* is 25, then, the minimum hash table size will be 2^50-33^ or 2^17^.

At this point, a brief discussion on the relation between the memory requirement and the quotient size is in order. We illustrate this using another example. Consider the case when we have 20 bits for the quotient value. Then for *k* = 32, the minimum hash table size is 2^44^ (2^64-20^). This will clearly affect the total memory requirement adversely. In fact, if we reduce the number of quotient bits by one, the minimum table size will be multiplied by two; on the other hand, increasing it will result in fewer hash table entries. Naturally, fewer entries in the hash table force more use of the auxiliary vector structures thereby increasing the running time. As it turns out, keeping 33 bits for the quotient value makes the right compromise: the memory requirement and the running time remain at an acceptable level and we can handle up to 32-mers.

#### 2.2.4. Hash Function Considerations

In our implementation, the hash values are 64-bit unsigned integers. We need 2*k* bits to represent a *k*-mer and *k* = 32. Theoretically, the hash value indices will never collide if the hash table consists of 2^64^ entries. But it is not practical to have a hash table of that size. So, we consider a much smaller hash table and then use multiple hash functions in order to reduce the probability of collision, filling as much space in the hash table as possible. So, it is mandatory to keep track of which hash function has been used for which *k*-mer.

Now, it seems that if we increase the number of hash functions, we can populate the hash table more efficiently. But there is a cost for storing the index of the hash function used for a particular *k*-mer. Clearly, if we allocate *n* bits for storing the hash function's index, then 2*^n^* the number of hash functions can be used. In our implementation using 5 bytes, we allocate 33 bits to store the quotient and 4 bits for the next nucleotide(s). So, we can allocate the remaining 3 bits for the index value of the hash function. So, we can use up to 2^3^ = 8 different hash functions.

### 2.3. The Procedure

**Figure 2:**
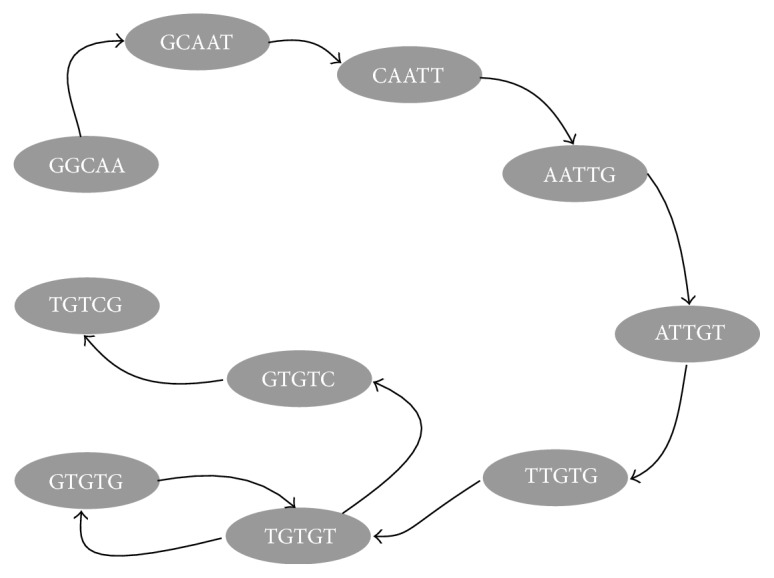
The de Bruijn graph for the *k*-mers. Nodes are GGCAA, GCAAT, CAATT, AATTG, ATTGT, TTGTG, TGTGT, GTGTG, TGTGT, and GTGTC.

To understand the whole process, here we explain how HaVec works with the help of an illustrative example. In this example, we assume that the values of *k* and *h* are 5 and 2, respectively. First, we consider a read from an input file that can be in FASTA or FASTQ format. We generate the *k*-mers from the read and compute the corresponding hash values for each *k*-mer. Now assume that we have *GGCAATTGTGTGTCG* as a read sequence from the input file. We will have to work on 5-mers and use 2 different hash functions. Clearly, we get the following 5-mers: *GGCAA*, *GCAAT*, *CAATT*, *AATTG*, *ATTGT*, *TTGTG*, *TGTGT*, *GTGTG*, *TGTGT*, *GTGTC*, and *TGTCG*.  Figure 2 illustrates the de Bruijn graph constructed using the above 5-mers.

**Figure 3:**
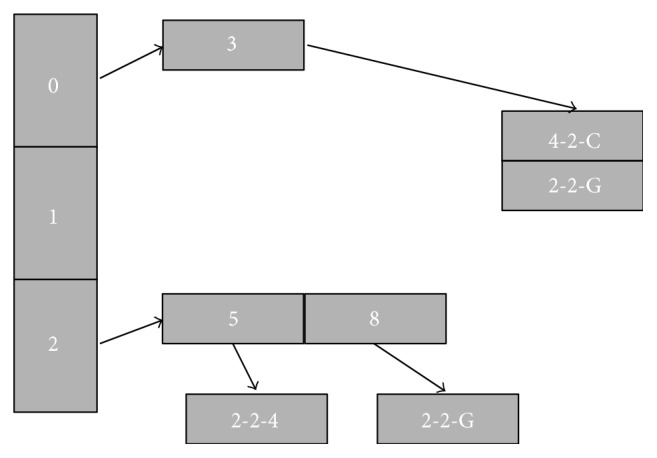
Auxiliary vector data structures. All collided *k*-mers' information of the hash table index 3 can be found at vector index 0, and all collided *k*-mers' information of hash table index 5 and index 8 can be found at vector index 2.

For the sake of ease of explanation, let us assume that the hash table size is 11. Suppose the two hash functions we have are *hash*1 and *hash*2. Due to brevity, we only report the hash values for each *k*-mer in Table 2 skipping any detail. Initially, each entry of the hash table is free; this is indicated by 0-0. The following format is used to store a *k*-mer's information in a hash table entry: (*quotient-which_hash_function-neighbour_info*). The information of each 5-mer in our example is reported in Table 3.  Figure 3 illustrates the way we handle the case of collided 5-mers.

We consider the first 5-mer, namely, *GGCAA*. As has been reported in Table 2, for *GGCAA*, *hash*1 returns 57. Since the hash table size is 11, we easily calculate the index to be 2 (57%11). So, the quotient is 5 ((*int*)(57_11)) and we further store *which_hash_function = 1*. Moreover, we need to set *neighbour_info = T* because by appending “T” with the suffix of *GGCAA*, we get its only neighbour in the de Bruijn graph, namely, *GCAAT* (see Figure 2). The same procedure is repeated for all of the successive 5-mers.

Now, let us focus on the proceedings related to *CAATT*. For this 5-mer, *hash*1 returns 24 generating again an index value of 24%11 = 2, which is already in use (due to *GGCAA*). So, we employ *hash*2 and it returns 36. This results in a different index, namely, 3 (36%11 = 3). Since this is a free index, we can safely put the information of *CAATT* here with 2 as the value of *which_hash_function*.

For the next 5-mer, namely, *ATTGT*, both *hash*1 and *hash*2 return already occupied indices. So here, the auxiliary vector data structure comes to the rescue. For the Level 1 vector, we use the index generated by *hash*2, that is, 5. In our example, we have assumed that the size of the first level vector is 3. So, we calculate 2 (5%3) to be the index for the first level vector. Recall that, since more than one hash table indices can point to the same index in the vector structure, HaVec maintains a list of entries for these different hash table indices. For this case, we create a new entry 5 at index 2 of the auxiliary vector data structure. All the collided *k*-mers for this index (i.e., the hash table index 5) will be stored in a separate 3rd level vector, which is pointed to from here. In particular, here we store the information (2–2–*G*) corresponding to the *k*-mer *ATTGT* at the 3rd level vector. The readers are kindly referred to Figure 3 for better understanding.

The handling of *TTGTG* is identical to that of *GGCAA*. *TGTGT* is handled in the same way as *ATTGT*. Both *hash*1 and *hash*2 return already occupied indices. *hash*2 returns 8. We divide it by the vector size (3) to get 2 as the remainder. Hence, a new entry 8 is created at index 2 of the level 1 vector. Clearly, all the collided *k*-mers for index 8 will now be stored in the 3rd level vector which is pointed to from here. Hence, we store (2–2–*G*).

The next *k*-mer, *GTGTG* is handled the same way as *ATTGT* and *TGTGT*. At this point again, we are faced with *TGTGT*. As it is already stored in our auxiliary vector structure, we simply need to update the neighbour's information. We simply add *C* as its next neighbour.

The last *k*-mer is *TGTCG*. For this one, *hash*1 returns 56, and we get 56%11 = 1 as the index in the hash table, which is a free index. Hence, we put this 5-*mer* related information there easily.

### 2.4. Cutoff Value and a 6-Byte Structure

In genome assembly, *cutoff value* is a threshold to determine the validity of a *k*-mer. In particular, a genome assembler will ignore a *k*-mer if it appears less frequently than the preset cutoff value.

Notably, the issue of a cutoff value has become less significant in recent times than it was before few years ago. This is because of the rapid advancement of the technologies in the sequencing laboratories that are now able to produce very high-quality reads much accurately. This motivated us not to keep provisions for a cutoff value in our original design. However, HaVec can easily accommodate cutoff values simply by using an additional byte. This allows us to support cutoff values between 1 and 255. When we process the input file, we can easily update the count information of a *k*-mer while updating its neighbour information. Subsequently, during the assembly process, the count can be easily compared with the preset cutoff value to decide on the validity of a *k*-mer. In HaVec implementation, we have parameterized the cutoff calculation. In the usual case, HaVec uses 5 bytes to store the *k*-mer information as opposed to 6 bytes in the implementation with provisions for cutoff values. This results in lower memory consumption as well as lower running time. In the rest of this paper, these two different implementations are referred to as the 5-*byte* and 6-*byte* implementations.

## 3. Results

To evaluate the performance of HaVec, we have conducted extensive experiments. We have run our experiments on a server with an Intel® Xeon® CPU E5-4617 @ 2.90 GHz having 12 cores with a total RAM of 64 GB. Note that the scope of this research was to implement HaVec as a single thread, and hence we have used only one core of the server for our experiments. We do plan to release a multithreaded version of HaVec in the near future.

Table 4 briefly describes the datasets we have used in our experiments. Notably, the datasets listed in Serial numbers 1, 2, and 3 in Table 4 have also been used by [7] in their experiments. All the data files in FASTA format can be downloaded from the following link: https://drive.google.com/drive/folders/0B3D-hZtRZ933SzgyVzc5Z2hUVkE?usp=sharing. Note that the illumina datasets (i.e., Serial numbers 2 to 4) are available in the BAM format. Therefore, BamTools (https://github.com/pezmaster31/bamtools) has been used to convert these files to FASTA format.

**Figure 4:**
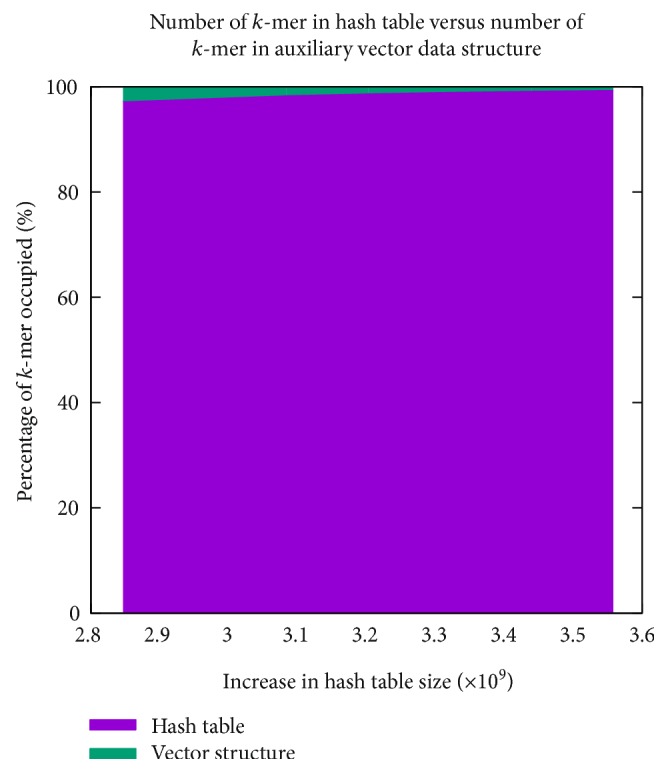
The relation between the number of *k*-mers in the hash table and the number of *k*-mers in the vector structure on increasing hash table size. A total of 19 cases are reported in the *x*-axis, where hash table size of case *i* + 1 is 5% higher than case *i* (1 ≤ *i* ≤ 18). As the hash table size increases, the number of *k*-mers in the hash table increases and the number of *k*-mers in the vector structure decreases. The same relation with the exact same values holds for both of the 5-byte and 6-byte implementations.

We first have designed an experiment with a goal to understand and analyze the relation among different parameters of HaVec. This experiment is done on the input file 50 m.fa assuming *k* = 27. It may be noted here that [8] also considered *k* = 27 in their experiments. The results have been presented in Figures 4(), 5(), 6(), and 7(). In Figure 4(), the relation between the number of *k*-mers in the hash table and in the vector data structure has been manifested. In particular, Figure 4() reports a total of 19 cases where case *i* + 1 assumes its hash table size to be 5% higher than that of case *i* (1 ≤ *i* ≤ 18). From Figure 4(), we notice a certainly desirable property of HaVec: an increase in the hash table size and a decrease (increase) in the number of *k*-mers in the vector structure (hash table). Notably, the same relation holds for both 5-byte and 6-byte implementations of HaVec and that too with the exact same values.

**Figure 5:**
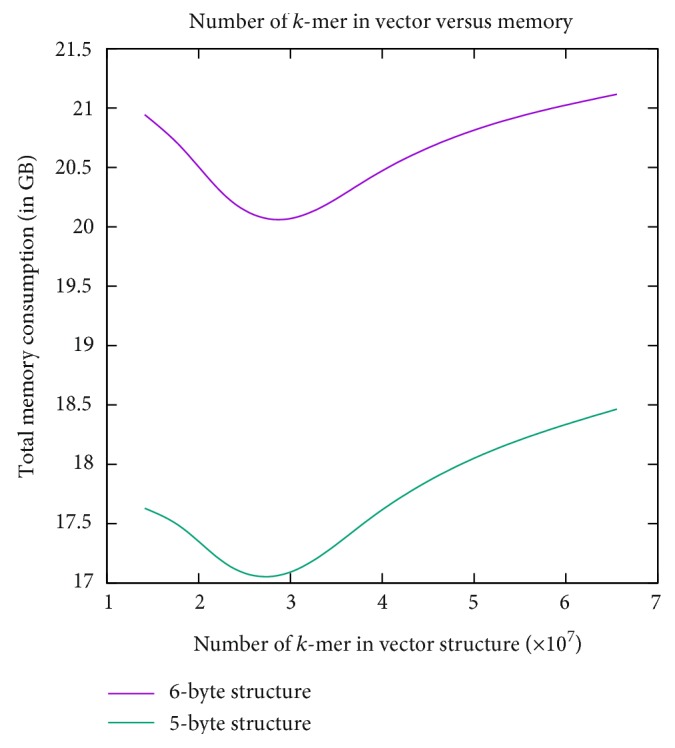
The graph shows the relation between the number of *k*-mers in the vector and the total memory. As the number of *k*-mers in the vector structure increases, the total memory also increases.

Next, we investigate the relation between the number of *k*-mers in the vector and the total memory. The results are depicted in Figure 5. As is evident from the figure, with the increase of *k*-mers in the vector structure, the total memory also increases. Also, as is evident from the difference between the two curves, the difference in memory requirements between the 5-byte and 6-byte implementations always remains constant.

**Figure 6:**
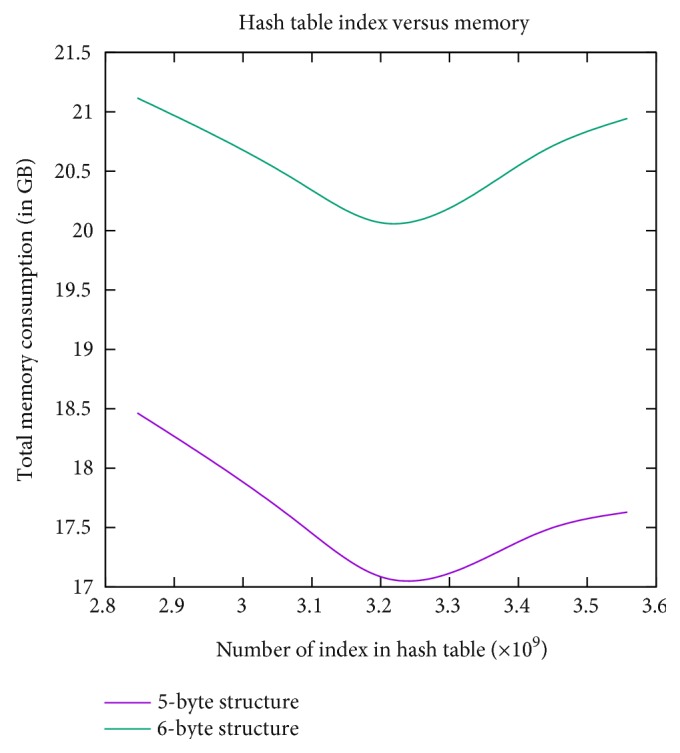
The relation for hash table index and total memory. Optimum memory use can be achieved when hash table size is 1.25 to 1.5 times the number of unique *k*-mers.

Figure 6 shows the curve for hash table size versus total memory, which sheds some light on how total memory changes with the increasing hash table size. As can be seen, larger hash table size does not always guarantee lower total memory consumption. Our experiments suggest that optimum memory use is achieved with a hash table size that is 1.25 to 1.5 times the number of unique *k*-mers.

**Figure 7:**
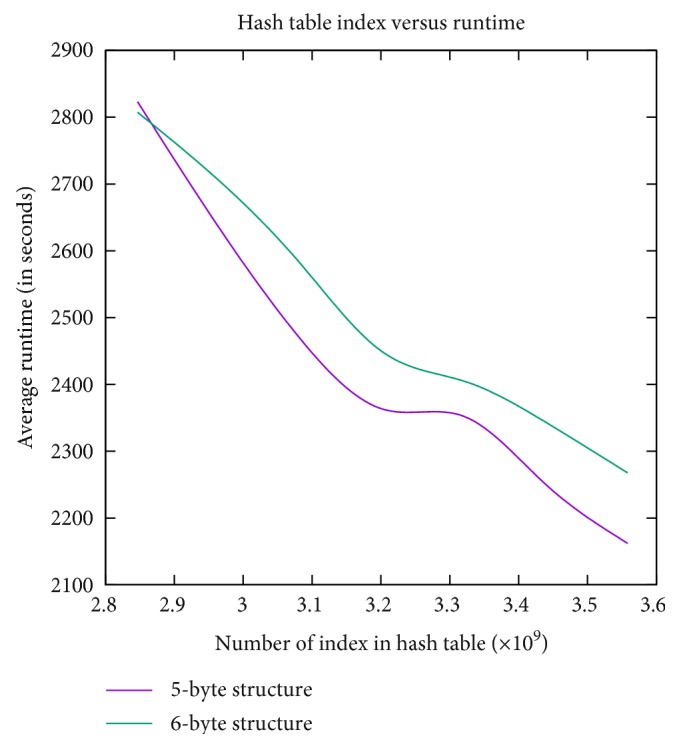
The relation between the hash table size and the runtime. Generally, runtime for the 6-byte implementation is slightly higher than that of the 5-byte implementation.

Finally, the curve in Figure 7 is for hash table size versus runtime. As can be seen from the figure, the runtime decreases with the increase in the hash table size. A final observation is that the running time for the 5-byte implementation is slightly lower than that of the 6-byte implementation.

We have further conducted extensive experiments considering all the files listed in Table 4. We have conducted these experiments for two different values of *k*, namely, 27 and 32. The results are presented in Table 5, where the hash table size, running time, total memory usage, total unique *k*-mers in the input file, and the number of *k*-mers in the hash table and in the vector data structures are reported. We have considered both 5-byte and 6-byte implementations of HaVec while reporting the total memory usage and the running time.

### 3.1. Comparison

We have conducted a number of experiments to compare the performance of HaVec with the state of the art methods. In particular, we have compared HaVec with Velvet [10] and minia [8]. We have used the file 50 m.fa for this comparison. The dataset in 50 m.fa is a soil metagenomics dataset. This MSB2soil dataset is available as SRA accession SRA050710.1. During our experiments, Velvet could not complete the processing of this file even after two hours of running even with 64 GB of RAM as the total memory got exhausted just after two hours. On the other hand, for minia, we have found that the running time is dependent on the free hard disk space. In particular, for minia, the free hard disk space has been found to be inversely proportional to the running time. We have used the following command to run minia: ./minia 50 m.fa 27 1 4500000000 output.

This command runs minia for *k* = 27 with a cutoff value of 1. With this command, the *k*-mer generation stage in minia has taken approximately 9.8 hours using a total of 59.5 GB of hard disk space. On the contrary, HaVec takes only 35.3 minutes using only 17.1 GB of RAM to produce *k*-mers along with their neighbour information which are completely error-free.

It should be mentioned here that in our experiments, minia has produced approximately 5% less unique *k*-mers than HaVec. Also, this percentage increases with the increase in the cutoff value. In genome assembly, generally, more unique *k*-mers are desirable as they produce longer output contig. The runtime of HaVec is independent of any nonzero cutoff value, and HaVec in fact runs faster with no cutoff value.

For minia, we have run the experiments for both values of *k*, that is, 27 and 32. The results are reported in Table 6. On the other hand, Velvet was run only for 27 (i.e., *k* = 27) and the results are reported in Table 7. For both of these experiments, consumed memory and runtime are reported in the corresponding tables. We have only considered Velvet [10] and minia [8] because they are reported to have performed better than other genome assemblers like SPAdes [11] and ABySS [4]. Notably, our method is not comparable with the Jellyfish algorithm [12] as it is a multithreaded approach and only counts the occurrences of *k*-mers.

### 3.2. Statistical Tests

We have conducted *t*-test to check whether the performance of HaVec over minia is statistically significant. The results are reported in Table 8. Here, we have documented 14 test runs, two for each data set. For *t*-test, the degree of freedom is defined as the *number of sample* − 1 which is 14 − 1 = 13 in this case. So, the degree of freedom is equal to 13 (df = 13). In bioinformatics, 95% confidence interval is used normally. To be stated as significant in terms of *t*-test, the *t*-value must be greater than 2.16 for df = 13, CI = 95%. As can be seen from Table 8, the improvements achieved are clearly statistically significant.

## 4. Discussion

In this paper, we have presented HaVec, which is a simple and efficient approach to store a de Bruijn graph for genome assembly. HaVec uses hash table along with an auxiliary vector data structure to store *k*-mers and their neighbouring information. One of the startling feature of HaVec is that it is completely error free as it does not generate any false positives.

HaVec can also support the concept of cutoff values by storing the count information of each *k*-mer. This count information can be compared to a preset cutoff value to filter *k*-mers at a later stage.

Any operation involving a *k*-mer in HaVec (i.e., insert, search, update, and remove) can be done by virtually in constant time as discussed below. Each of these operations has the same time complexity of *O*(*n* + *m*) when applied on the auxiliary vector structure, where *n* and *m* refer to the number of collided hash table indices at a vector structure index and the number of collided *k*-mers at a hash table index, respectively. Clearly, the value of *n* is very small, because most of the *k*-mers are stored in the hash table. In fact, in our experiments, we have always found *n* ≤ 4. Another important parameter is the ratio of the number of *k*-mers in the vector data structure to the number of *k*-mers in the hash table. And in our experiments, the average value of this parameter has been found to be 1/1000 which is extremely low. So, any insert or information retrieval can be done virtually in constant time, which makes HaVec a really fast de Bruijn graph construction and information retrieval process.

Before concluding, we briefly discuss another useful feature of HaVec. During assembly, the construction of the de Bruijn graph and the assembly process may need to be run more than once for different cutoff values. On the contrary, in the 6-byte implementation of HaVec, we just keep the count of the number of occurrences of each *k*-mer independent of any preset cutoff value. So, HaVec needs to construct a de Bruijn graph just once as opposed to multiple times in other methods like Velvet and minia where independent multiple runs are required for different cutoff values. More specifically, any graph which is already constructed using any of these methods for a particular cutoff value cannot be used for an assembly that requires a different cutoff value.

## 5. Conclusion

The major share of the time in the genome assembly process is taken by the graph construction procedure. In this paper, we have presented HaVec which can do this in a significantly shorter time. Another critical feature of HaVec is that it does not produce any false positive *k*-mers thereby making the graph error free. We anticipate that HaVec will be used by researchers and practitioners alike in bioinformatics and computational biology. Because parallelization of the de Bruijn approach has already been attempted in the literature (e.g., [13]), an immediate avenue for future research would be to see whether we can parallelize our approach by using multithreading concepts.

## Figures and Tables

**Algorithm 1:**
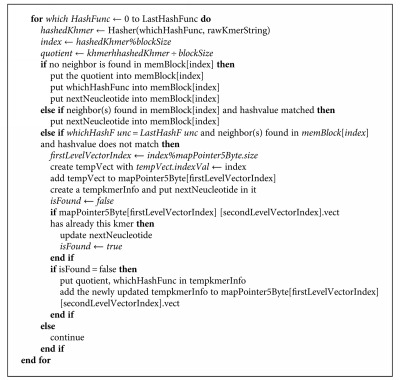
Formal steps of the algorithm.

**Table 1:** HaVec's usage of 5 bytes.

Information	Number of bits
Outgoing neighbours	4
Hash function number	3
Quotient value	33

**Table 2:** Reads are broken into *k*-mers. All *k*-mer's hash values are shown here.

*k*-mers	Hash values	Comments
GGCAA	57	
GCAAT	27	
CAATT	24, 36	
AATTG	52	
ATTGT	36, 27	Put in vector
TTGTG	22	
TGTGT	34, 30	Put in vector
GTGTG	49, 47	Put in vector
TGTGT	34, 30	Found in vector; update
GTGTC	38, 25	Put in vector
TGTCG	56	

**Table 3:** Hash table information. We have a total of 11 indices. At each index, the corresponding *k*-mer's quotient, hash function, and its neighbour information are saved.

Index	Information
0	2–1–*T*
1	5–1
2	5–1–*T*
3	3–2–*G*
4	0–0
5	2–1–*T*
6	0–0
7	0–0
8	4–1–*T*
9	0–0
10	0–0

**Table 4:** Description of datasets.

Serial number	File name	File size in MB
1	50 m.fa	030.42
2	Ecoli_MG1655_s_6_1_bfast.fasta	242.19
3	Ecoli_MG1655_s_6_2_bfast.fasta	1718.37
4	Human1_95G_CASAVA1.8a2_NCBI37_18Jan11_chr21.sorted.fasta	1599.86
5	Human1_95G_CASAVA1.8a2_NCBI37_18Jan_chr19.sorted.fasta	2393.17
6	NA19240_GAIIx_100_chr21.fasta	1854.83
7	dataset_1_7GB.fa	1677.57
8	dataset_1_9GB.fa	1944.92

**Table 5:** Results for HaVec.

File name	*k*	Hash table index	6 bytes per *k*-mer runtime (sec)	5 bytes per *k*-mer runtime (sec)	6 bytes per *k*-mer total memory (MB)	5 bytes per *k*-mer total memory (MB)	Unique *k*-mer in hash table	Unique *k*-mer in vector	Total unique *k*-mer
50 m.fa	27	3,558,218,093	2261.75	2163	21445.9392	18052.608	2,358,010,004	14,135,390	2,372,145,394
50 m.fa	32	3,283,745,651	2183	2038.5	19228.8768	16648.4992	2,176,128,060	13,035,689	2,189,163,749
Ecoli_MG1655_s_6_1_bfast.fasta	27	20,115,587	70.25	70	144.896	125.7472	13,330,622	79,768	13,410,390
Ecoli_MG1655_s_6_1_bfast.fasta	32	20,693,341	69.375	68.75	148.3776	128.6144	13,713,606	81,955	13,795,561
Ecoli_MG1655_s_6_2_bfast.fasta	27	196,614,919	488.5	486.5	1205.8624	1018.368	130,293,923	782,686	131,076,609
Ecoli_MG1655_s_6_2_bfast.fasta	32	200,937,899	480.75	476.5	1231.872	1040.2816	133,158,739	799,832	133,958,571
Human1_95G_CASAVA1.8a2_NCBI37_18Jan11_chr19.sorted.fasta	27	313,251,713	601.5	593.25	1909.0432	1610.24	207,579,166	1,255,278	208,834,444
Human1_95G_CASAVA1.8a2_NCBI37_18Jan11_chr19.sorted.fasta	32	334,345,241	611.625	592.75	2035.6096	1716.736	221,565,835	1,330,984	222,896,819
Human1_95G_CASAVA1.8a2_NCBI37_18Jan11_chr21.sorted.fasta	27	199,165,411	371.5	370.625	1221.4272	1031.4752	131,981,455	795,486	132,776,941
Human1_95G_CASAVA1.8a2_NCBI37_18Jan11_chr21.sorted.fasta	32	207,852,223	374.75	374.5	1273.4464	1075.2	137,741,484	826,661	138,568,145
NA19240_GAIIx_100_chr21.fasta	27	163,949,171	508.625	513.5	1009.5616	853.1968	108,643,167	656,266	109,299,433
NA19240_GAIIx_100_chr21.fasta	32	170,662,721	549	504.625	1016.3712	886.9888	113,094,644	680,508	113,775,152
dataset_1_7GB.fa	27	199,165,411	395.25	368.125	1221.4272	1031.4752	131,981,455	795,486	132,776,941
dataset_1_7GB.fa	32	207,852,223	373.75	367	1273.4464	1075.2	137,741,484	826,661	138,568,145
dataset_1_9GB.fa	27	163,949,171	516	507.125	1009.5616	853.1968	108,643,167	656,266	109,299,433
dataset_1_9GB.fa	32	170,662,721	511.375	507.625	1049.8048	886.9888	113,094,644	680,508	113,775,152

**Table 6:** Results for minia.

File name	*k* size	Min abundance	Estimated genome size	Runtime (AVG)	Runtime (SD)	Memory total (AVG)	Memory total (SD)
50 m.fa	27	1	2,189,163,749	37637.66666	1064.9044	6589.0	0
50 m.fa	32	1	2,189,163,749	33418.33333	477.8162	6604.0	0
Ecoli_MG1655_s_6_1_bfast.fasta	27	1	13,795,561	100	1.0000	251.0	0
Ecoli_MG1655_s_6_1_bfast.fasta	32	1	13,795,561	100.66667	1.5275	251.0	0
Ecoli_MG1655_s_6_2_bfast.fasta	27	1	133,958,572	1787	35.7911	1813.0	0
Ecoli_MG1655_s_6_2_bfast.fasta	32	1	133,958,572	1753	13.0767	1814.0	0
Human1_95G_CASAVA1.8a2_NCBI37_18Jan11_chr19.sorted.fasta	27	1	222,896,820	2229.66667	21.9393	2551.0	0
Human1_95G_CASAVA1.8a2_NCBI37_18Jan11_chr19.sorted.fasta	32	1	222,896,820	2075	7.5498	2553.0	0
Human1_95G_CASAVA1.8a2_NCBI37_18Jan11_chr21.sorted.fasta	27	1	138,568,143	1083.33333	8.5049	1697.0	0
Human1_95G_CASAVA1.8a2_NCBI37_18Jan11_chr21.sorted.fasta	32	1	138,568,143	1116.33333	15.1767	1698.0	0
NA19240_GAIIx_100_chr21.fasta	27	1	113,775,137	836.33333	7.5719	1935.0	0
NA19240_GAIIx_100_chr21.fasta	32	1	113,775,137	870.33333	9.0185	1935.0	0
dataset_1_7GB.fa	27	1	138,568,143	1084	10.1489	1697.0	0
dataset_1_7GB.fa	32	1	138,568,143	1103.66667	6.6583	1698.0	0
dataset_1_9GB.fa	27	1	113,775,137	841	9.5394	1935.0	0
dataset_1_9GB.fa	32	1	113,775,137	864.66667	7.5719	1935.0	0

**Table 7:** Results for Velvet.

File name	Disk space (GB)	RAM space	Run time (seconds)	Number of *k*-mers
50 m.fa	6.6	64,675,804 KB	83,900	50,000,000+
Ecoli_MG1655_s_6_1_bfast.fasta	1.9	751,460 KB	53	2,003,258
Ecoli_MG1655_s_6_2_bfast.fasta	13	5,884,072	411	14,214,324
Human1_95G_CASAVA1.8a2_NCBI37_18Jan11_chr19.sorted.fasta	7.3	6,591,012	327	17,670,833
Human1_95G_CASAVA1.8a2_NCBI37_18Jan11_chr21.sorted.fasta	5.0	4,107,472	188	11,812,904
NA19240_GAIIx_100_chr21.fasta	7.0	3,714,472 KB	246	15,016,990
dataset_1_7GB.fa	5.0	4,108,000 KB	187	11,812,904
dataset_1_9GB.fa	7.0	3,714,480 KB	242	15,016,990

**Table 8:** *t*-test results excluding 50 m.fa.

File name	Minia runtime	HaVec 5-byte mf = 1.2	HaVec 5-byte mf = 1.5	HaVec 6-byte mf = 1.2	HaVec 6-byte mf = 1.5
Ecoli_MG1655_s_6_1_bfast.fasta	100.00	86.625	0	85.875	70.25
Ecoli_MG1655_s_6_1_bfast.fasta	100.50	86.125	8.75	86.875	69.375
Ecoli_MG1655_s_6_2_bfast.fasta	1801.350	597.5	86.5	601.25	488.50
Ecoli_MG1655_s_6_2_bfast.fasta	1756.00	589.875	476.5	592.75	480.75
Human1_95G_CASAVA1.8a2_NCBI37_18Jan_chr19.sorted.fasta	2236.00	724.25	593.25	722	601.50
Human1_95G_CASAVA1.8a2_NCBI37_18Jan_chr19.sorted.fasta	2071.50	726.25	592.75	738.50	611.625
Human1_95G_CASAVA1.8a2_NCBI37_18Jan11_chr21.sorted.fasta	1087.50	449.5	370.625	444.125	371.50
Human1_95G_CASAVA1.8a2_NCBI37_18Jan11_chr21.sorted.fasta	1124.50	447.875	374.50	450.625	374.75
NA19240_GAIIx_100_chr21.fasta	832.00	621	513.50	623.625	508.625
NA19240_GAIIx_100_chr21.fasta	870.00	619.25	504.625	622.375	525.75
dataset_1_7GB.fa	1078.50	446.5	368.125	450.25	395.25
dataset_1_7GB.fa	1102.00	451.25	367	454.25	373.75
dataset_1_9GB.fa	841.50	619.875	507.125	628.375	516.0
dataset_1_9GB.fa	862.00	626	507.625	624.125	511.375
*t*-value	*NA*	*5.6466*	*5.1002*	*4.6367*	*5.0702*

## Data Access

All the data files in FASTA format can be downloaded from the following link: https://drive.google.com/drive/folders/0B3D-hZtRZ933SzgyVzc5Z2hUVkE?usp=sharing. The implementation of the proposed algorithm is hosted in the following repository: https://github.com/ratulSharker/Havec.
